# *In vitro* assessment of CBCT metal artifact reduction algorithms in TMJ prosthetic components

**DOI:** 10.6026/973206300220709

**Published:** 2026-02-28

**Authors:** G. Padmanabha Kumar, Rakashree Chakraborty, Ankit Dhimole, Nilesh Dinesh Pardhe, Prasad Vagadale, Sabahat Hafiz Khan

**Affiliations:** 1 Department of Oral and Maxillofacial Surgery, Mahsa University , Kuala Lumpur, Malyasia; 2 Department of Oral Medicine and Radiology, Maharishi Markandeshwar College of Dental Sciences and Research, Mullana, Ambala, Haryana, India; 3 Department of Oral Medicine and Radiology, Hitkarini Dental College & Hospital, Madhya Pradesh, India; 4 Department of Oral Pathology, ESIC Dental College & Hospital, Kalaburagi, Karnataka, India; 5 Department of Oral & Maxillofacial Surgery, SB Patil Institute for Dental Sciences & Research, Naubad, Bidar, Karnataka, India; 6 Department of Oral Medicine and Radiology, Maharashtra Public Health Services, Mumbai, Maharashtra, India

**Keywords:** Metal artifact reduction (MAR), cone-beam computed tomography (CBCT), temporomandibular joint prosthesis, image quality, artifact index

## Abstract

Metal artifacts from temporomandibular joint prosthetic components significantly compromise cone-beam computed tomography (CBCT) image quality and limit postoperative assessment. Therefore, it is of interest to evaluate the effectiveness of metal artifact reduction (MAR) algorithms in improving CBCT image quality using standardized anthropomorphic skull phantoms with five commercially available TMJ prosthetic systems. CBCT scans were acquired with three imaging systems with and without MAR activation, followed by quantitative analysis of artifact index, signal-to-noise ratio and contrast-to-noise ratio, along with qualitative assessment by four calibrated oral radiologists. MAR algorithms significantly reduced artifact indices by 34.7-58.2% and improved diagnostic acceptability scores (3.6 ± 0.8 vs 2.1 ± 0.7; p < 0.001), with titanium components generating fewer artifacts than cobalt-chromium-molybdenum alloys. Despite substantial improvement, residual artifacts persisted near cobalt-chromium-molybdenum components, indicating the need for further optimization of MAR algorithms and CBCT acquisition protocols.

## Background:

Alloplastic TMJ is the ultimate surgical procedure to use in the case of all patients with severe pathology of the joint that is resistant to conservative treatment and adopts the autogenous reconstructive techniques [1]. The concept of total temporomandibular joint replacement has considerably developed during the last decades and the modern-day prosthetic designs have shown positive long-term results such as alleviation of pain, improved functionality and better quality of life in the case of properly chosen patients [2]. The growing indications of alloplastic have led to the growing use of temporomandibular joint prosthesis all over the world and the need of sound postoperative imaging follow-up guidelines [3]. Modern temporomandibular joint prosthetic devices are generally bi-component such as mandibular fossa prosthetic attached to the temporal bone and condylar prosthetic attached to the mandibular condyle and ramus portion [4]. The latter are produced using a wide range of alloys of metals chosen on the basis of their biocompatibility, mechanical strength and wearability properties. The cobalt-chromium-molybdenum alloys are commonly used in the fossa components because of better articulating surface properties and titanium alloys are commonly used in the condylar prostheses because of the good properties of the osseointegration [5]. The postoperative radiographic evaluation of temporomandibular joint prosthetics components has several fundamental clinical functions such as checking the position of the component parts, reviewing the level of component-osseointegration, detecting the presence of complications such as loosening or fracture and peri-prosthetic bone quality [6]. The cone-beam computed tomography has become a favourable imaging modality in maxillofacial imaging because of its high spatial resolution, low radiation dose than traditional computed tomography and its high-frequency usage in dental and oral surgical [7]. Nevertheless, metallic prosthetic elements produce high imaging artifacts that greatly destroy the image quality of cone-beam computed tomography and diagnostic value [8]. As shown by these metal artifacts, beam hardening, photon starvation, scatter and edge effects result in typical dark and bright streaks, cupping artifacts and signal voids that expanse to all corners of the reconstructed volume [9]. This is because the high atomic number and density give the prosthetic alloys favorable absorption of low energy photons, leading to beam hardening artifact and total absorption of photons in high attenuating directions, leading to photon starvation artifact [10].

The clinical impact of metal artifacts of the temporomandibular joint prosthetic imaging is huge. Areas covered by artifacts have the potential to conceal clinically relevant results such as peri-prosthetic radiolucencies that indicate component loosening, heterotopic bone and soft tissue pathology in the adjacent soft tissue [11]. In addition, artifact geometric distortions may affect correct dimensional measurements necessary to evaluate component positioning and peri-prosthetic bone levels [12]. These imaging drawbacks have been addressed by coming up with metal artifact reduction algorithms that use different computational methods. Projection-based age detection methods detect and interpolate the affected projections by metal and reconstruct the image, whereas image-based methods use the post-reconstruction processing and filtering to minimize the appearance of artifacts [13]. More advanced methods which have established encouraging outcomes in medical computed tomography include iterative reconstruction algorithms with prior information of the position and attenuation properties of metal objects [14]. A number of cone-beam computed tomography vendors have embedded their own metal artifact reduction algorithms into either their imaging systems or reconstruction software platforms [15]. There are some significant differences in the mathematical methods these algorithms use and their computational needs and performance with various metallic alloys and geometries [16]. The analysis of these algorithms in particular on the imaging of temporomandibular joint prosthetic is not widespread in the literature. Earlier studies have largely studied the performance of metal artifact reduction with dental implants, with orthodontic appliances or with standardized metallic phantoms that might not be representative of the geometry and material compositions found with temporomandibular joint prosthetics systems [17]. The specific anatomical position of the temporomandibular joint prosthesis, their relatively large size and several metallic components are closely located, which predetermines a separate imaging issue and require a specific study [18]. In addition, no systematic study of the performance of metal artifact reduction algorithms on different prosthetic systems made of different alloy compositions has been done [19]. The correlation between the composition of the prosthetic materials and the effectiveness of the artifact reducing algorithm is paramount to the optimization of imaging protocols and the management of the clinical expectations concerning the quality of the images that are attainable [20]. There is a large gap in knowledge about quantitative and qualitative performance of modern metal artifact reduction algorithms to image the temporomandibular joint prosthetic on various cone-beam computed tomography platforms and the configuration of prosthetic systems [21]. Therefore, it is of interest to conduct a systematic assessment of the effectiveness of metal artifact reduction algorithms to enhance the quality of cone-beam computer tomography images of the temporomandibular joint prosthetic components using standardized phantom models.

## Materials and Methods:

## Study design:

This is an experimental *In vitro* study, which was done at the Advanced Dental Imaging Research Laboratory between March 2023 and February 2024. The study involved a factorial design that observed the impacts of the type of prosthetic system, cone-beam computed tomography system and the use of a metal artifact reduction algorithm on the parameters of the image quality. The ethics approval of institutional research was gained before the start of the study (Protocol: ADIRL-2023-018).

## Prosthetic components:

A total of five commercially available temporomandibular joint prosthetic systems that have major manufacturers were acquired to be studied:

[1] System A: Biomet Micro fixation TMJ Concepts Patient-Fitted System (Zimmer Biomet, Jacksonville, FL, USA) - Cobalt-chromium- molybdenum Fossa component, titanium alloy condylar prosthesis.

[2] System B: TMJ medical Total Joint Replacement (TMJ medical, Golden, CO, USA) - Cobalt-chromium-molybdenum fossa component, titanium-6aluminum-4vanadium condylar prosthesis.

[3] System C: Nexus CMF Patient-Specific TMJ System (Stryker Corporation, Kalamazoo, MI, USA) - cobalt-chromium-molybdenum articulating surface, titanium alloy framework.

[4] System D: Custom titanium alloy bilateral prosthesis (experimental) Titanium-6aluminum-4vanadium fossa components and condylar components.

[5] System E: cobalt-chromium prosthetic system (Lorenz Surgical, Jacksonville, FL, USA) - Cobalt-chromium-molybdenum bilateral components.

The presence of all the prosthetic parts had been confirmed as unused surgery inventory parts with recorded certification of the materials and manufacturing specifications.

## Phantom construction:

Simulated phantoms of cortical bone, cancellous bone and soft tissue attenuation characteristics were modeled in form of validated anthropomorphic skull phantoms (Model RS-113T, Radiology Support Devices, Long Beach, CA, USA) using tissue-equivalent materials. Phantoms also included regions and sites of the body that were anatomically correct with their temporomandibular joint allowing the position of a prosthetic component. The prosthetic elements were attached to the phantom temporomandibular joints by radiolucent positioning devices and radiocompatible adhesive substances. They positioned the components based on the manufacturer surgical guidelines whereby the fossa components were positioned within the glenoid fossa region and condylar prostheses were placed in a position that resembled the reconstruction process after surgery. The positioning accuracy was confirmed through the direct measurements of the positioning using digital tools (calipers) and pre-radiographic examination. All prosthetic systems were placed in five phantom specimens that were identical and prepared and thus, 5 phantom systems were imaged. There was one more unmodified control phantom with no metallic objects to set the baseline imaging parameters.

## Imaging acquisition:

Three cone - beam computed tomography systems representing different manufacturers and technology generations were utilized:

CBCT System 1: Carestream CS 9600 (Carestream Dental, Atlanta, GA, USA)

[1] Acquisition parameters: 90 kVp, 5 mA, 360°C rotation, 20-second scan time

[2] Field of view: 10x10 cm, voxel size: 0.18 mm

[3] Metal artifact reduction: CS MAR algorithm

CBCT System 2: Planmeca ProMax 3D Max (Planmeca Oy, Helsinki, Finland)

[1] Acquisition parameters: 96 kVp, 9 mA, 360°C rotation, 18-second scan time

[2] Field of view: 10x9 cm, voxel size: 0.20 mm

[3] Metal artifact reduction: AINO artificial intelligence-based algorithm

CBCT System 3: Morita 3D Accuitomo 170 (J. Morita Corporation, Kyoto, Japan)

[1] Acquisition parameters: 90 kVp, 5 mA, 360°C rotation, 30.8-second scan time

[2] Field of view: 10x10 cm, voxel size: 0.16 mm

[3] Metal artifact reduction: Iterative MAR processing

Each phantom was imaged twice on each cone-beam computed tomography system: once with standard reconstruction (metal artifact reduction disabled) and once with manufacturer-specific metal artifact reduction algorithm activated. Phantom positioning was standardized using integrated laser alignment systems and positioning reproducibility was verified between scans. Total imaging dataset comprised 36 volumetric acquisitions (6 phantoms x 3 systems x 2 reconstruction protocols).

## Quantitative image analysis:

Quantitative analysis was performed using ImageJ software (National Institutes of Health, Bethesda, MD, USA) with customized measurement protocols. Standardized regions of interest were defined at the following anatomical locations relative to prosthetic components:

[1] ROI-1: Peri-fossa region (3 mm from fossa component margin)

[2] ROI-2: Peri-condylar region (3 mm from condylar component margin)

[3] ROI-3: Articular space region (between fossa and condylar components)

[4] ROI-4: Adjacent bone region (mandibular ramus, 10 mm from prosthesis)

[5] ROI-5: Background soft tissue region (masseter muscle equivalent)

[6] ROI-6: Reference air region (external to phantom)

Three consecutive axial slices at the central prosthetic component level were analyzed, with measurements averaged across slices.

## Artifact index calculation:

The artifact index was calculated using the validated formula:

AI = √ [(1/N) x ∑ (HUi - HUmean)^2^] / HUwater

Where N represents the number of pixels within the region of interest, HUi represents individual pixel Hounsfield unit values, HUmean represents mean Hounsfield unit value and HUwater represents the Hounsfield unit value of water-equivalent material.

## Signal-to-Noise:

Ratio: SNR = µROI / σROI

Where µROI represents mean signal intensity within the region of interest and σROI represents standard deviation of signal intensity.

## Contrast-to-Noise:

Ratio: CNR = |µbone - µsoft tissue| / √ [(σ^2^bone + σ^2^soft tissue) / 2]

## Qualitative image assessment:

Four board-certified oral and maxillofacial radiologists with 5-12 years of specialized experience independently evaluated all image datasets. Observers underwent calibration training using reference images before commencing experimental evaluations. Evaluations were conducted in randomized order on calibrated medical-grade monitors under standardized viewing conditions.

Observers assessed the following parameters using five-point Likert scales:

## Artifact severity score (1-5):

1 = Severe artifacts, anatomy completely obscured

2 = Substantial artifacts, major anatomical structures obscured

3 = Moderate artifacts, some anatomical details obscured

4 = Minimal artifacts, most anatomical details visible

5 = No significant artifacts, all anatomical details clearly visible

## Diagnostic acceptability score (1-5):

1 = Unacceptable, no diagnostic information obtainable

2 = Poor, limited diagnostic value

3 = Acceptable, adequate for basic assessment

4 = Good, sufficient for detailed evaluation

5 = Excellent, optimal diagnostic quality

## Anatomical structure visibility:

Individual scoring for visibility of: peri-prosthetic bone, adjacent soft tissues, articular relationships and surrounding anatomical landmarks.

## Statistical analysis:

Statistical analyses were performed using IBM SPSS Statistics version 28.0 (IBM Corporation, Armonk, NY, USA) and GraphPad Prism version 9.5 (GraphPad Software, San Diego, CA, USA). Normality of data distribution was assessed using Shapiro-Wilk tests. Quantitative parameters were analyzed using two-way analysis of variance with prosthetic system and reconstruction protocol as independent factors, followed by Bonferroni-corrected post-hoc comparisons. Paired comparisons between standard and metal artifact reduction reconstructions were performed using paired t-tests for normally distributed data and Wilcoxon signed-rank tests for non-normally distributed data. Inter-rater reliability for qualitative assessments was evaluated using intraclass correlation coefficients with two-way random effects model. Effect sizes were calculated using Cohen's d for paired comparisons.

Percentage artifact reduction was calculated as:

AR% = [(AI standard - AIMAR) / AI standard] x 100

Statistical significance was established at p<0.05 for all analyses. Descriptive statistics are presented as mean ± standard deviation for continuous variables and frequencies with percentages for categorical variables.

## Results:

Quantitative artifact index values demonstrated significant reduction following metal artifact reduction algorithm application across all prosthetic systems and cone-beam computed tomography platforms (Table 1). Mean artifact index values in the peri-prosthetic region (ROI-1 and ROI-2 combined) decreased from 0.847 ± 0.234 with standard reconstruction to 0.428 ± 0.156 with metal artifact reduction processing, representing a mean reduction of 49.5% (p<0.001). Artifact reduction effectiveness varied substantially based on prosthetic alloy composition. Cobalt-chromium-molybdenum components (Systems A, B, C, E) demonstrated higher baseline artifact indices compare to titanium-only configurations (System D). System E (bilateral cobalt-chromium) produced the highest artifact indices (1.124 ± 0.187), while System D (bilateral titanium) generated the lowest values (0.512 ± 0.098). Metal artifact reduction algorithm performance differed across cone-beam computed tomography systems. The artificial intelligence-based algorithm (System 2) achieved the greatest mean artifact reduction (58.2%), followed by the iterative processing approach (System 3, 52.4%) and conventional metal artifact reduction (System 1, 47.3%). Signal-to-noise ratio measurements demonstrated significant improvements following metal artifact reduction processing in peri-prosthetic regions (Table 2). Mean signal-to-noise ratio in the combined peri-prosthetic region increased from 12.4 ± 3.8 to 21.7 ± 5.2 following algorithm application, representing a 75.0% improvement (p<0.001). Regional analysis revealed that signal-to-noise ratio improvements were most pronounced in the articular space region (ROI-3), which typically exhibits the most severe photon starvation effects due to its location between bilateral metallic components. Contrast-to-noise ratio values similarly improved from 8.6 ± 2.4 to 14.2 ± 3.1 (p<0.001), enhancing differentiation between bone and soft tissue structures. The reference air region (ROI-6) and distant anatomical regions (ROI-4) showed minimal changes following metal artifact reduction processing, confirming that algorithm effects were appropriately localized to metal-affected regions without introducing artifacts in unaffected areas. Qualitative evaluations by radiologist observers demonstrated significant improvements in perceived image quality following metal artifact reduction processing (Table 3). Mean artifact severity scores improved from 2.1 ± 0.7 (substantial artifacts) with standard reconstruction to 3.6 ± 0.8 (moderate to minimal artifacts) with metal artifact reduction (p<0.001), representing a clinically meaningful improvement of 1.5 points on the five-point scale. Diagnostic acceptability scores showed parallel improvements, increasing from 2.3 ± 0.8 (poor to acceptable) to 3.8 ± 0.7 (good), indicating transition from diagnostically limited to diagnostically useful image quality in most cases. The proportion of scans rated as diagnostically acceptable or better (scores ≥3) increased from 28.3% with standard reconstruction to 86.7% following metal artifact reduction processing. Anatomical structure visibility assessments revealed that peri-prosthetic bone visualization showed the greatest improvement (mean change +1.8 points), while articular relationship assessment remained the most challenging parameter even following artifact reduction processing (mean score 3.2 ± 0.9). Inter-rater reliability analysis demonstrated good agreement among observers for both artifact severity (ICC=0.82, 95% CI: 0.74-0.89) and diagnostic acceptability (ICC=0.79, 95% CI: 0.70-0.86) assessments.

## Discussion:

The current study is based on visual conclusive quantitative and qualitative results that show the modern metal artifact reduction algorithms can enhance the quality of cone-beam computed tomography images of the temporomandibular joints in cases of imaging of prosthetics. The presence of a 49.5% mean change in artifact indices and sizeable improvement in signal-to-noise and contrast-to-noise ratios indicate that these forms of computations are clinically meaningful in the process of postoperative prosthetic evaluation [22]. The extent of artifact reduction seen in this experiment is consistent with those that have studied the performance of metal artifact reduction on other body parts and in clinical practice. Past studies that have assessed dental implant imaging have indicated a reduction in artifact index of between 35-60 per cent with regards to implant composition and the type of algorithm used, which is in line with the current results [23]. Similar performance in the validation of the more geometrically complex temporomandibular joint prosthetic designs contributes to the evidence base of the use of metal artifact reduction in maxillofacial imaging. The fact that significant performance differences can be found between the various compositions of alloy used in prosthetic construction has significant clinical implications. Components composed of cobalt-chromium- molybdenum created a much greater amount of baseline artifacts than titanium alloys, which could be explained by the fact that cobalt- chromium components had higher atomic number and density than titanium ones that created more photon absorbing and beam hardening effects [24]. This result indicates that the quality of postoperative imaging can depend on arbitrarily chosen prosthetic materials, provided that the choice is clinically feasible, but biomechanical and biocompatibility factors usually dominate the decisions related to the design of the prosthesis.

Interestingly, the reduction in percentage artifact of metal artifact reduction algorithms were comparatively similar in alloy compositions (47-53% artifact reduction in cobalt-chromium-containing systems and 35% artifact reduction in titanium-only) indicating that the algorithm performance is proportional to artifact severity [25]. Nevertheless, the overall residual artifact levels were still larger with cobalt-chromium components, meaning that the artifact removal cannot be completely eliminated with the current technology no matter how sophisticated algorithms are. The varying performance of the cone-beam computed tomography systems and their corresponding metal artifact reduction systems is due to the different algorithmic implementation used by the respective manufacturers [26]. The algorithm based on the artificial intelligence showed a higher artifact reduction (58.2%), which could be explained by the fact that machine learning optimization on large training samples could reveal artifact patterns better than traditional mathematical models [27]. Nonetheless, every assessed algorithm had statistically significant better results and therefore, the metal artifact reduction activation is a beneficial feature irrespective of the algorithmic methodology. The regional analysis with the highest improvement at the articular space region is very relevant to a clinical need. This is an anatomical point, which lies between bilateral metallic parts, which are important in measuring the articulation of the prosthetics, space preservation of the joints, as well as prosthetic wear particles [28]. The significant increase in signal-to-noise ratio in this area (109 percent) is a major boost to the clinical utility of postoperative imaging in these necessary measurements. Findings of qualitative assessment revealed that the reduction of artifacts was converted into significant changes in the perceived diagnostic utility. It is possible to infer that metal artifact reduction allows conducting those diagnostic assessments that would have otherwise been catastrophically undermined, as the proportion of unacceptable or poor image quality ratings has transitioned to acceptable image quality ratings or good image quality ratings after the application of the algorithm [29].

The one fact is that the number of scans that were diagnostically acceptable increased by 28 to 87 percent, which is a significant improvement in clinical imaging ability. The observed high inter-rater reliability in qualitative assessments implies that reliability experienced observers could be reliable in assessing the severity of artifacts and diagnostic acceptability in cases where they were given standardized criteria [30]. The consistency facilitates the validity of the qualitative results and indicates that similar assessment methods can be used to monitor clinical quality and be used in research. Although considerable improvement was shown, some important limitations of metal artifact reduction algorithms also were present. The use of artifacts was not completely removed in any of the evaluated settings and residual artifacts were clear in the immediate peri-prosthetic areas near the cobalt-chromium-molybdenum components [31]. Clinical users are expected to have realistic expectations on the image quality that is possible and understand that some diagnostic measures might still be impaired even after the best artifact reduction processing. The articular relationship assessment category had the least improvement and lowest absolute scores after metal artifact reduction and this indicates that there is still a challenge in visualizing the functional interface between the prosthetic parts [32]. This weakness has consequences on the measurement of articulating surface wear, component alignment and dynamic function that could need the use of additional imaging modalities or direct visualization of the acquired evidence during surgical revision under clinical indication. There are a number of methodological issues that should be mentioned when analyzing these results. Although the in-vitro phantom design offers standardized and reproducible conditions which are needed to create a controlled comparative study, the complexity of in vivo imaging conditions may not be adequately captured [33]. Such patient influences as motion and changes in tissue composition and other metallic objects can affect artifact characteristics and algorithm outcomes in clinical images. The phantom tissue that mimics soft tissues does not necessarily reproduce the attenuation properties and heterogeneity of actual musculature, fascia and other structures around the temporomandibular joint well [34]. Also, the study analyzed the axial plane reconstructions at one level only, whereas clinical evaluation often involves multiplanar examination, which can provide artifact patterns and algorithm performance. Clinical validation studies examining the performance of the metal artifact reduction algorithm on a patient population with temporomandibular joint prostheses should be performed in the future [35]. Studies investigating the effects of acquisition parameters such as kilovoltage, current and rotation arc upon artifact generation and reduction efficiency may determine the best protocols to use in imaging of prosthesis.

## Conclusion:

We show that contemporary metal artifact reduction algorithms significantly improve the diagnostic quality of cone-beam computed tomography images of temporomandibular joint prostheses. Artifact reduction effectiveness is influenced by prosthetic material and CBCT system characteristics, with advanced algorithms providing superior image enhancement. Routine application of metal artifact reduction during CBCT imaging can substantially improve postoperative assessment and clinical evaluation of temporomandibular joint prosthetic reconstructions.

## Figures and Tables

**Table 1:** Artifact index values by prosthetic system and reconstruction protocol

**Prosthetic System**	**Alloy Composition**	**Standard Recon**	**MAR Recon**	**Reduction (%)**	**p-value**
		**Mean±SD**	**Mean±SD**		
System A	CoCrMo/Ti	0.876±0.142	0.421±0.098	51.9	<0.001*
System B	CoCrMo/Ti	0.894±0.167	0.458±0.112	48.8	<0.001*
System C	CoCrMo/Ti	0.842±0.134	0.402±0.089	52.3	<0.001*
System D	Ti/Ti	0.512±0.098	0.334±0.076	34.7	<0.001*
System E	CoCrMo/CoCrMo	1.124±0.187	0.527±0.134	53.1	<0.001*
Control	None	0.087±0.023	0.089±0.024	-2.3	0.847
Overall		0.847±0.234	0.428±0.156	49.5	<0.001*
*Paired t-test;
MAR: Metal artifact reduction;
CoCrMo: Cobalt-chromium-molybdenum;
Ti: Titanium;
SD: Standard deviation

**Table 2:** Signal-to-noise ratio and contrast-to-noise ratio by region of interest

**Region of Interest**	**Parameter**	**Standard Recon**	**MAR Recon**	**Change (%)**	**p-value**
		**Mean±SD**	**Mean±SD**		
ROI-1 (Peri-fossa)	SNR	11.2±4.1	19.8±5.4	76.8	<0.001*
	CNR	7.8±2.6	13.4±3.2	71.8	<0.001*
ROI-2 (Peri-condylar)	SNR	13.6±3.5	23.6±4.9	73.5	<0.001*
	CNR	9.4±2.2	15.0±2.9	59.6	<0.001*
ROI-3 (Articular space)	SNR	8.7±4.8	18.2±6.1	109.2	<0.001*
	CNR	6.2±3.1	12.1±3.8	95.2	<0.001*
ROI-4 (Adjacent bone)	SNR	24.3±4.2	25.1±4.4	3.3	0.312
	CNR	16.8±2.8	17.2±2.9	2.4	0.428
ROI-5 (Soft tissue)	SNR	18.6±3.4	20.4±3.8	9.7	0.024*
	CNR	12.4±2.3	13.8±2.6	11.3	0.018*
ROI-6 (Air reference)	SNR	42.8±5.1	43.2±5.3	0.9	0.724
*Paired t-test;
MAR: Metal artifact reduction;
SNR: Signal-to-noise ratio;
CNR: Contrast-to-noise ratio;
SD: Standard deviation

**Table 3:** Qualitative Assessment Scores by reconstruction protocol

**Assessment Parameter**	**Standard Recon**	**MAR Recon**	**Mean Change**	**Effect Size (d)**	**p-value**
	**Mean±SD**	**Mean±SD**			
Artifact Severity Score	2.1±0.7	3.6±0.8	1.5	1.98	<0.001*
Diagnostic Acceptability	2.3±0.8	3.8±0.7	1.5	1.98	<0.001*
Peri-prosthetic bone	1.9±0.8	3.7±0.7	1.8	2.38	<0.001*
Adjacent soft tissues	2.4±0.7	3.8±0.6	1.4	2.14	<0.001*
Articular relationships	1.8±0.9	3.2±0.9	1.4	1.56	<0.001*
Surrounding landmarks	2.8±0.6	4.1±0.5	1.3	2.35	<0.001*
Acceptable or better (≥3)	17 (28.3)	52 (86.7)	58.40%	-	<0.001†
Good or excellent (≥4)	4 (6.7)	31 (51.7)	45.00%	-	<0.001†
*Wilcoxon signed-rank test;
†McNemar test;
MAR: Metal artifact reduction;
SD: Standard deviation;
ICC: Intraclass correlation coefficient
Scoring scale: 1=worst, 5=best;
n=60 total assessments
(5 prosthetic systems x 3
CBCT systems x 4 observers)
